# Combining anterior and posterior component separation for extreme cases of abdominal wall reconstruction

**DOI:** 10.1007/s10029-020-02152-3

**Published:** 2020-03-05

**Authors:** J. Lopez-Monclus, J. Muñoz-Rodríguez, C. San Miguel, A. Robin, L. A. Blazquez, M. Pérez-Flecha, N. Rupealta, M. A. Garcia-Urena

**Affiliations:** 1 Henares University Hospital (Coslada, Madrid), Faculty of Health Sciences, Francisco de Vitoria University, Carretera Pozuelo-Majadahonda km. 1,800, 28223 Pozuelo de Alarcón, Spain; 2 Puerta de Hierro University Hospital, Majadahonda, Madrid, Spain; 3 Ramón y Cajal University Hospital, Madrid, Spain

**Keywords:** Transversus abdominis release, Anterior component separation, Synthetic mesh, Complex hernia, Posterior component separation

## Abstract

**Purpose:**

The closure of midline in abdominal wall incisional hernias is an essential principle. In some exceptional circumstances, despite adequate component separation techniques, this midline closure cannot be achieved. This study aims to review the results of using both anterior and component separation in these exceptional cases.

**Methods:**

We reviewed our experience using the combination of both anterior and posterior component separation in the attempt to close the midline. Our first step was to perform a TAR and a complete extensive dissection of the retromuscular preperitoneal plane developed laterally as far as the posterior axillary line. When the closure of midline was not possible, an external oblique release was made. A retromuscular preperitoneal reinforcement was made with the combination of an absorbable mesh and a 50 × 50 polypropylene mesh.

**Results:**

Twelve patients underwent anterior and posterior component separation. The mean hernia width was 23.5 ± 5. The majority were classified as severe complex incisional hernia and had previous attempts of repair. After a mean follow-up of 27 months (range 8–45), no case of recurrence was registered. Only one patient (8.33%) presented with an asymptomatic bulging in the follow-up. European Hernia Society’s quality of life scores showed a significant improvement at 2 years postoperatively in the three domains: pain (*p* = 0.01), restrictions (*p* = 0.04) and cosmetic (*p* = 0.01).

**Conclusions:**

The combination of posterior and anterior component separation can effectively treat massive and challenging cases of abdominal wall reconstruction in which the primary midline closure is impossible to achieve despite appropriate optimization of surgery.

## Introduction

Incisional hernias (IH) are a frequent complication after midline laparotomies. A number of IH are considered complex because of size, location, domain loss, previous operations, the presence of stomas or infection, and comorbidities [1, 2]. The main goal of surgical repair of midline IH is to obtain a complete fascial approximation, with an appropriate mesh reinforcement.

However, some midline IH have extensive defects, thus for midline advancement of very widely separated borders, different component separation techniques have been described, and are divided into two groups: anterior component separation (ACS) and posterior component separation (PCS). Albanese first described ACS by releasing the insertion of external oblique muscles [3]. Ramirez implemented this technique in a cadaver study, which combined medial posterior rectus sheath release with external oblique detachment [4]. Later, this technique was effectively used with mesh reinforcement [5–7]. ACS generated advancements as long as 10 cm. However, a main issue with this technique was morbidity associated with the wound, due to extensive subcutaneous detachment. To avoid these complications, modifications have been described for ACS, e.g. laparoscopic ACS and perforator preserving ACS [8, 9].

The PCS technique emerged only in the last decade; the approach facilitated closure of the midline with an extended lateral dissection, as in the previously described retromuscular preperitoneal plane [10]. The first PCS study was published in 2008, and described lengthening the lateral dissection between the internal oblique and transversus abdominis muscles [11]. After the initial publication of the transversus abdominis release (TAR) [12], this PCS approach spread rapidly among abdominal wall reconstruction (AWR) surgeons [6, 13–16], as it avoids subcutaneous dissection of ACS, and dissects a landing zone in a retromuscular position to lay the mesh as a reinforcement. The Madrid modification of the TAR was also implemented without a need for cutting the transversus abdominis (TA) muscle and reinserting the transversus muscle into the mesh [17].

In some challenging cases, despite using the component separation technique, there may still be difficulties in reintroducing the viscera and closing the midline. Also, abdominal closure may induce development of intra-abdominal hypertension and respiratory distress [18, 19]. In these instances, quilted mesh, bridging repairs, or visceral resections have been performed [16, 20, 21]. Bridged repairs are associated with a high risk of recurrence [21]. In our experience, intestinal resection and anastomosis carry a risk of anastomotic dehiscence that may be fatal for the patient. Therefore, we decided to combine ACS and PCS for exceptional circumstances.

The aim of this study is to present data on TAR and ACS combinatorial approaches during exceptional and challenging cases, to achieve AWR.

## Materials and methods

We performed a retrospective study on patients undergoing hernia repair at two specialized complex hernia repair centers, by reviewing a prospective AWR multicenter database. Patients undergoing AWR using a PCS approach, and those who also required an additional ACS for midline closure, where identified and reviewed. Ethical approval was obtained from the Institutional Review Board before study commencement. The report was written following the STROBE statement [22], and the recommendations for reporting outcomes in abdominal wall hernias [23].

All patients were evaluated in a multidisciplinary unit, specialized in abdominal wall procedures. Collected measures included age, sex, body mass index (BMI), comorbidities, type of hernia according to the European Hernia Society (EHS) classification, number of previous hernia repairs, and details of the initial operation. Surgical data included length of surgical procedures, width and length of the abdominal wall defect and other required surgical procedures (adhesiolysis, bowel resection, panniculectomy). In the postoperative period, any systemic complications or surgical site events were recorded, e.g. length of hospital stay.

All patients followed a similar preoperative optimization program, which included endocrinological and nutritional evaluations, smoking abstinence, weight loss and respiratory physiotherapy. Injection of botulinum toxin into the lateral abdominal wall musculature was performed under ultrasound guidance, approximately 4 weeks before surgery, as previously described [24]. This technique was applied to hernia defects longer than 10 cm width, started in 2017. Progressive pneumoperitoneum, with daily insufflation of 500 ml of air, was performed during the two weeks before surgery, for cases with > 25% Tanaka index [25].

## Surgical technique

**Fig. 1:**
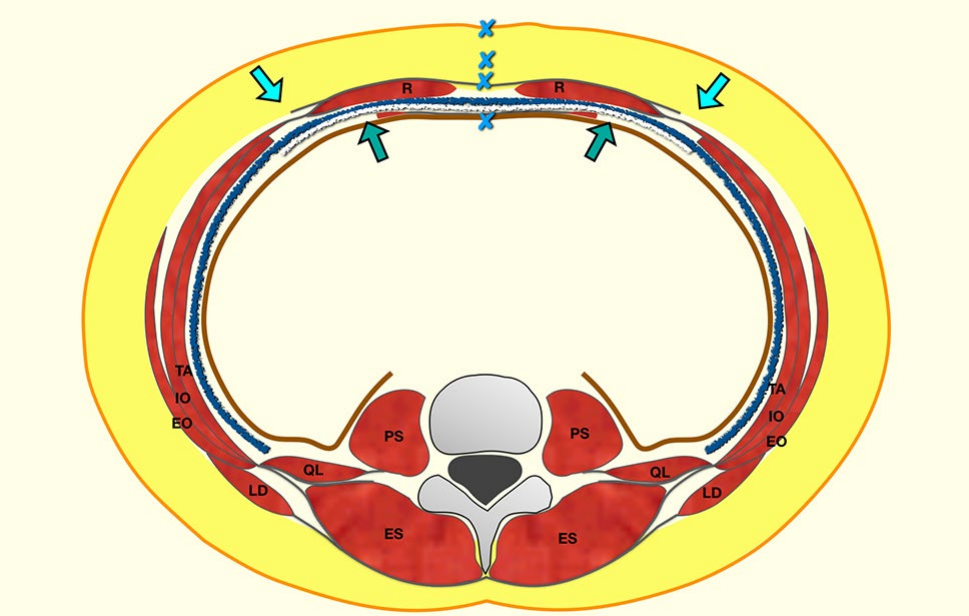
Schematic representation of AWR with the combination of ACS and PCS. Absorbable mesh is depicted with a white line and polypropylene mesh with a blue line. The big synthetic mesh overlaps both weakened surfaces created by the external oblique and transversus abdominis releases. *EO* external oblique muscle, *IO* internal oblique muscle, *TA* transversus abdominis muscle, *R* rectus muscle, *PS* psoas muscle, *QL* quadratus lumborum, *LD* latissimus dorsi, *ES* erector spinea muscles. Blue line: polypropylene mesh; white line: absorbable mesh; blue X: lines of sutures

A schematic representation of the surgical technique is shown (Fig. 1). Under general anesthesia, the patient was placed in decubitus supinus, with a bladder catheter for intravesical pressure measurements, and an epidural catheter for pain management. The skin was incised through the previous scar, removing any unaesthetic scars, if present. If the patient had a large skin apron, the procedure was performed through a low transverse incision between both anterosuperior iliac spines, lifting all subcutaneous tissue until the epigastric area was reached. The hernia sac was opened longitudinally in two halves, and the bowel and omentum content were reduced. Half the hernia sac was left attached to the anterior rectus sheath, and the other half was left attached to the contralateral posterior rectus sheath. A meticulous adhesiolysis was performed if there were any adhesions to the hernia sac, or the parietal peritoneum. The abdominal content was then protected with a towel.

The posterior rectus sheath was opened bilaterally, and a retromuscular dissection was performed, reaching both the lateral borders of the posterior rectus sheath, as the lateral limit of the dissection. Perforator neurovascular bundles were visualized and carefully preserved. Midline cranial dissection was performed, raising the undamaged linea alba by cutting and pulling down both posterior rectus sheaths and preperitoneal fat in the midline, until the central tendon of the diaphragm is reached. Caudally, the retromuscular dissection was overextended to detect both the Cooper’s ligaments. At this time of the surgery, two stitches of long-term absorbable monofilament (Monomax^®^, USP 00, Mesh Elastic, B. Braun, Melsungen, Germany) were placed in both Cooper’s ligaments to fix the permanent mesh later.

Then, a down to up TAR was performed, as previously described [17]. The created lateral retromuscular preperitoneal space was extended laterally as far as the posterior axillary line, reaching the quadratus lumborum and psoas muscle. Whenever possible, we aimed to cut the posterior rectus sheath more medially, cranially, to avoid transecting of the TA muscle fibers.

The posterior layer, comprising the posterior rectus sheath and part of the attached sac, was approximated with a running suture of a long-term absorbable monofilament not in all but one case, in which this closure could not be initially performed. In this case, after the initial attempt to reintroduce the abdominal content, there was a high peak of respiratory pressure. After the additional release of the external oblique muscle, the respiratory parameters improved, enabling posterior closure.

For mesh reinforcement, we used an absorbable posterior reinforcement of permanent mesh in a complex hernia (the Madrid Approach) [16]. According to, a piece of a 20 × 30 cm absorbable mesh (GORE^®^ BIO-A^®^ Tissue Reinforcement, WL Gore & Associates, Inc. Flagstaff, AZ, USA) is placed over the posterior layer closure, to provide support for a large 50 × 50 cm polypropylene macroporous mesh (Bulevb^®^, Dipro Medical Devices SRL, Torino, Italy), which is fixed to the Cooper’s ligaments. As required, one or two 15F low-suction drains are placed over the mesh. The lateral border of the posterior rectus sheath is then reinserted into the permanent mesh, with running absorbable sutures.

At this point of the surgery, a first attempt to bring together the midline of the medial limits of the anterior rectus sheath is carried out. If closure of the anterior layer is impossible and the presence of an unacceptable gap according to the physical characteristic of the patient or in case of severe respiratory distress for the anesthesiologist, the decision to add an ACS is taken. The ACS is performed in two ways, depending on the initial approach. In cases where an extensive subcutaneous dissection has been performed (big and redundant subcutaneous sacs, with exposure of the anterior fascia further the lateral limit of the anterior rectus sheath, or an association of a panniculectomy), a fasciotomy is performed as described by Ramirez [4].

In case of no extensive lateral subcutaneous dissection, the ACS is performed in a minimally invasive open approach, as described [26]. Two 2 cm transverse skin incisions are performed at the point of the intersection of the umbilical line, and the mammarian line. The subcutaneous tissue is dissected until exposure of the external oblique aponeurosis. A small transverse incision is performed in this layer. With blunt blind dissection, the avascular space is dissected with the finger. A blind external oblique fasciotomy is performed, pushing partially closed long scissors in the cranial and caudal border of the aponeurosis opening, reaching both the costal margin and the inguinal ligament.

Once the ACS has been performed, the midline is closed with a running suture of long-term absorbable monofilament (Monomax^®^, USP 1 or O, Mesh Elastic, B.Braun, Melsungen, Germany). In case complete closure is not possible despite addition of ACS and PCS, the gap is bridged over the mesh, suturing the border of the anterior rectus sheath to the polypropylene mesh, with running sutures of long-term absorbable monofilaments (Monomax^®^, USP 00, Mesh Elastic, B.Braun, Melsungen, Germany). To isolate the exposed mesh from subcutaneous tissue, part of the hernia sac that remains attached to the anterior rectus sheath is sutured to the contralateral border of the posterior rectus sheath, with full coverage of the bridged mesh. After closure of the anterior layer, one or two low-suction drains are placed in the subcutaneous space, in case of an associated panniculectomy. The subcutaneous tissue is sutured with short-term absorbable stitches, and the skin is closed with staples.

## Follow-up

Follow-up evaluations consisted of a physical examination in the outpatient clinic at 4–5 weeks, 3 months, 6 months, 1 year, and then annually thereafter. Patients underwent an abdominal computed tomography (CT) scan in case of clinical doubts, or as part of oncological surveillance.

Long-term complications such as chronic pain, chronic mesh infection, recurrence, and bulging were evaluated at each visit. Recurrence was defined as a new abdominal wall defect in the operated area, as identified by physical examination or imaging. Bulging was defined as an area of weakness or asymmetry during patient abdominal wall inspection or exploration, without any solution of continuity in the abdominal wall visible by CT. We used the European Abdominal Wall Hernia Quality of Life Scores (EuraHS-QoL) to compare evolution in patients by pain, restriction and cosmetic domains between the preoperative and postoperative periods [27].

## Statistics

Variable descriptions and statistical analyses were performed using the Statistical Package for the Social Sciences (SPSS) program (version 19.0 for Windows). In an intention to perform the analysis, quantitative variables were expressed as the mean/median and standard deviation/quartiles, and categorical variables were expressed as absolute numbers and percentages. Spearman’s correlation coefficient was used to measure the strength of relationship between paired data of EuraHS-QoL scores.

## Results

### Patient demographics and characteristics

Between 2014 and 2019, 12 patients who underwent AWR, required combined PCS and ACS. Patient demographics and characteristics are shown (Table 1). The mean age was 62.66 ± 11.8 years, and the mean BMI was 37.19 ± 7.94 kg/m^2^. Eight patients had a previous history of incisional hernia repair.

**Table 1:** Demographics and characteristics of patients

Variables	*N* (%)
Sex	
Male	5 (41.7%)
Female	7 (58.3%)
Age, mean ± DS	62.66 ± 11.8
BMI^a^, mean ± DS	37.19 ± 7.94
Obesity (BMI > 30)	10 (83.33%)
Comorbidities	
Smoking Anticoagulation Diabetes Immunosuppression COPD^b^ Hypertension Neoplasia Cardiac disease	0 (0%) 0 (0%) 4 (33.3%) 1 (8.3%) 5 (41.7%) 6 (50%) 6 (50%) 1 (8.3%)
CeDAR^c^; mean ± DS	48.66 ± 17.44
< 30% 30–60% > 60%	2 (16.7%) 7 (58.33%) 3 (25%)
ASA^d^	
I II III IV	1 (8.3%) 6 (50%) 5 (41.7%) 0 (0%)
Recurrent	8 (66.6%)
Number of previous hernia repairs, median (min–max)	2 (0–5)
Etiology of main IH	
Digestive tube Urology Gynecology and obstetrics Others	6 (50%) 2 (16.7%) 2 (16.7%) 2 (16.7%)

^a^Body mass index

^b^Chronic obstructive pulmonary disease

^c^Carolinas equation for determining associated risks

^d^American society of anesthesiologists

### Hernia characteristics

Hernia features are summarized in Table 2. The mean width of the horizontal aponeurotic defect was 23.58 ± 4.91 cm, and the mean vertical length was 19.91 ± 5.50 cm. All defects were midline, ten (83.3%) were M1–M5 according to EHS classification, and two (16.7%) presented a concomitant lateral incisional hernia.

**Table 2:** Characteristics of IH

	*N* (%)
EHS classification of main IH	
Midline M1–M5 M3–M5	12 (100%) 10 (83.33%) 2 (16.7%)
EHS classification of associated IH	
Lateral L3	2 (16.7%) 2 (16.7%)
Maximum horizontal size cm; mean ± DS	23.58 ± 4.91
Maximum vertical size cm; mean ± DS	19.91 ± 5.50
W EHS	
W1 (< 4 cm) W2 (4–10 cm) W3 (> 10 cm)	0 (0%) 0 (0%) 12 (100%)
Slater’s classification	
Grade 1 Grade 2 Grade 3	0 (0%) 4 (33.3%) 8 (66.7%)
VHWG^a^ classification	
Grade 1 Grade 2 Grade 3 Grade 4	1 (8.3%) 10 (83.3%) 1 (8.3%) 0 (0%)
VHSS^b^ classification	
Grade 1 Grade 2 Grade 3	0 (0%) 3 (25%) 9 (75%)

^a^Ventral hernia working group hernia classification

^b^Ventral hernia staging system classification

### Operative details

Operative details are presented (Table 3). In seven cases (58.33%), preoperative pneumoperitoneum was performed with a median of 11.800 ml (5.000–12.000 ml) air. Botulin toxin injection was used in five cases (41.66%). Ten cases (83.33%) were classified as clean and clean-contaminated wounds. The posterior layer could be closed in all cases. Three patients (25%) required an anterior layer bridge, without the possibility of complete closure of the midline, with a mean horizontal bridge of 2.9 cm, and a mean vertical bridge of 3 cm. There was a 25% rate of enterotomies; all initially repaired. Associated surgeries included; omentum resection for two cases (16.7%), and in nine cases (75%), a panniculectomy was performed as part of the AWR.

**Table 3:** Operative data

Variables	*N* (%)
Wound classification [43]	
Clean Clean-contaminated Contaminated Dirty	9 (75%) 1 (8.3%) 2 (16.7%) 0 (0%)
Bridging of posterior layer	0 (0%)
Bridging of anterior layer	3 (25%)
Maximum diameter of bridging; mean (min–max)	
Horizontal Vertical	2.9 (0–7) 3 (0–8)
Associated surgery for the IH repair	
Adhesiolysis Omentum resection Closure of bowel opening Panniculectomy	7 (58.3%) 2 (16.7%) 3 (25%) 9 (75%)
Operative time (min), mean ± DS	339.16 ± 66.18

### Postoperative complications

Postoperative complications are summarized (Table 4). Surgical site infections (SSI) were reported in four cases (33.3%): superficial SSI in two patients (16.7%), deep SSI in two cases (16.7%), and no organ/space SSI was registered. The overall incidence of surgical site occurrences (SSO) was 66.6%. Seven cases (58.3%) developed a seroma in the first 30 postoperative days. In 25% of patients, a skin/wound dehiscence was registered. There were five SSO (41.7%) that required procedural interventions (SSOPI): four bed-side drains, and one chronic seroma that required reintervention with a talc seromadesis (28). In terms of systemic complications, there were three cases (25%) of pneumonia with an associated respiratory insufficiency, and one case required intensive care. No postoperative mortalities were registered. No patient developed intra-abdominal hypertension.

**Table 4:** Postoperative complications

Variable	*N* (%)	Clavien–Dindo
SSO		
Any SSO	8 (66.66%)	
SSOPI	5 (41.7%)	
SSI	4 (33.3%)	
Superficial Deep Organ/space	2 (16.7%) 2 (16.7%) 0 (0%)	Grade I: 2 (16.7%) bed-side treatments Grade II: 2 (16.7%) antibiotics + bed-side treatments
Hematoma	0 (0%)	
Seroma	7 (58.3%)	Grade II: 4 (33.3%) bed-side treatments + antibiotics treatment. Grade IIIb: 1 reintervention
Skin/wound dehiscence Fascial disruption	3 (25%) 0 (0%)	Grade I: 3 (25%)
Abdominal complications		
Paralytic ileus Anastomotic dehiscence	1 (8.3%) 0 (0%)	Grade I: 1 (8.3%) conservative treatment
Systemic complications		
Urinary infection	4 (33.3%)	Grade II: 4 (33.3%) antibiotics treatment
Venous line infection Respiratory insufficiency	3 (25%) 3 (25%)	Grade I: 3 (125%) removal of cathether Grade II: 2 (16.7%): antibiotics treatment. Grade IVa: 1 (8.3%) intensive care
Renal insufficiency	0 (0%)	
Pneumonia	3 (25%)	Grade II: 2 (16.7%): antibiotics treatment. Grade IVa: 1 (8.3%) Intensive care
Cardiac complications DVT/PE^a^	1 (8.3%) 0 (0%)	Grade I: 1 (8.3%) diuretic treatment
Pain > 48 h requiring opioids	8 (66.7%)	
Length of hospitalization, median, (min–max)	7 (1–54)	
30 day mortality	0 (0%)	
Readmission	3 (25%)	

^a^Deep venous thrombosis/pulmonary thromboembolism; α Intensive care unit

### Long-term postoperative complications

Long-term postoperative complications are shown (Table 5). After a mean follow-up of 27 months (range 8–45), no recurrences were registered. Only one patient (8.33%) presented with an asymptomatic bulging at follow-up. No cases of chronic pain or chronic mesh infection were identified. No mortalities were recorded during follow-up.

**Table 5:** Long-term postoperative complications

Variables	*N* (%)
Clinical recurrence	0 (0%)
CT control	
No CT performed No CT recurrence Yes CT recurrence	5 (41.7%) 7 (58.3%) 0 (0%)
Mesh infection	1 (0.8%)
Pain	
Discomfort Occasional need for pain treatment Daily treatment for pain Interventional treatment for pain; no pain	5 (41.7%) 1 (8.3%) 0 (0%) 0 (0%)
Bulging	
No bulging Asymptomatic bulging	11 (91.7%) 1 (8.3%)
Reoperation for recurrence or bulging	0 (0%)

### Quality Of Life

**Fig. 2:**
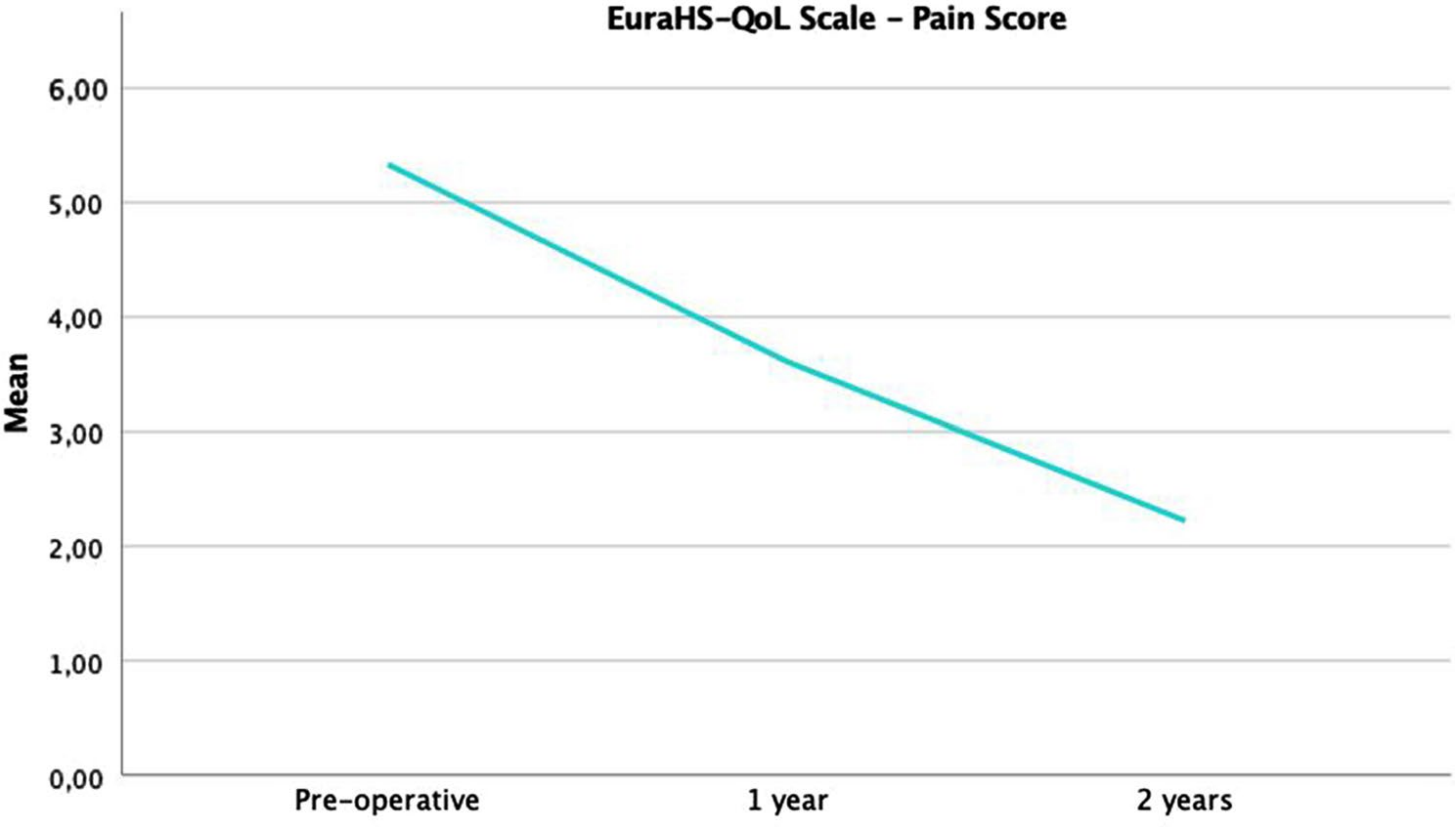
The evolution over time of EuraHS-QoL of pain domain is shown

**Fig. 3:**
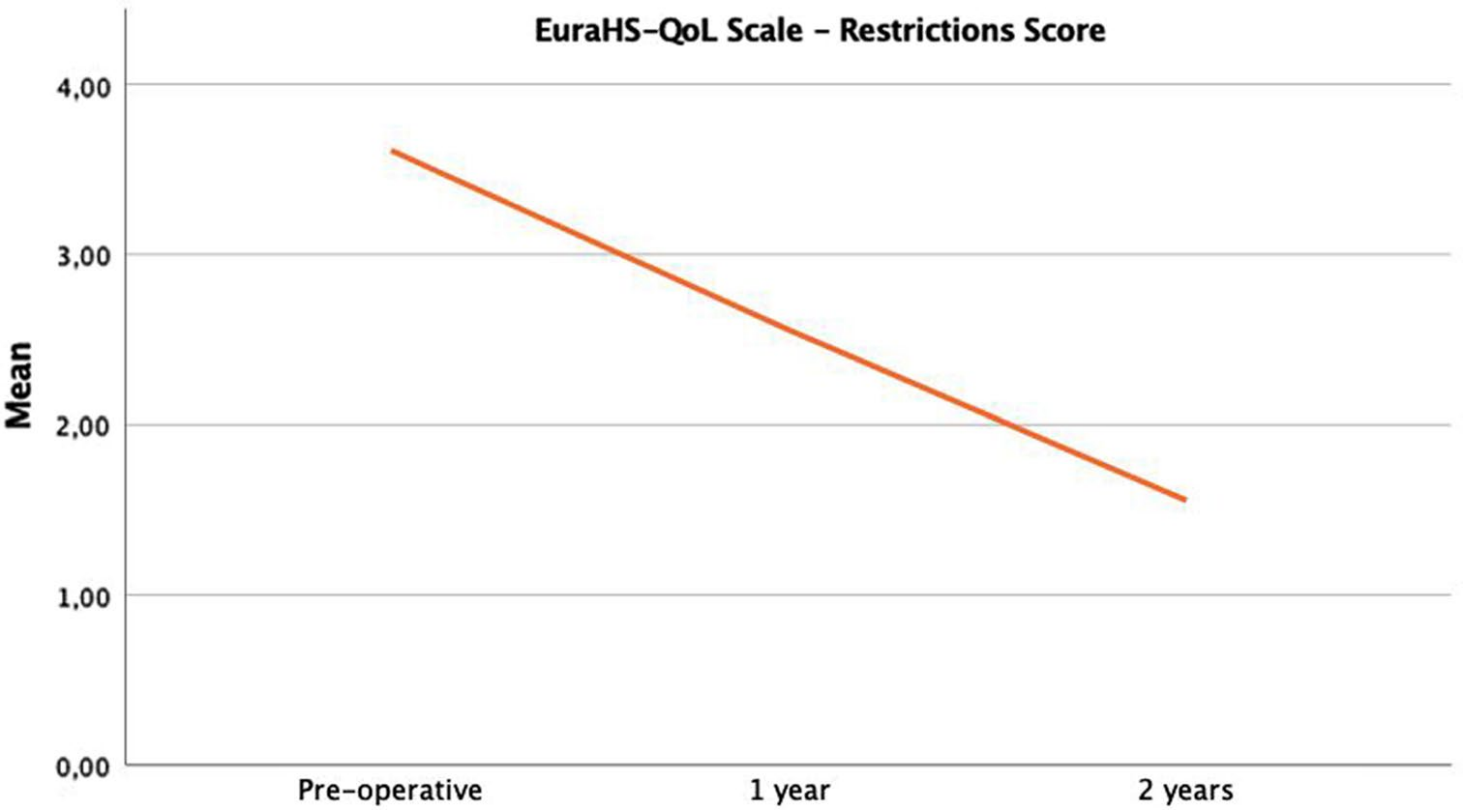
The evolution over time of EuraHS-QoL of restrictions domain is shown

**Fig. 4:**
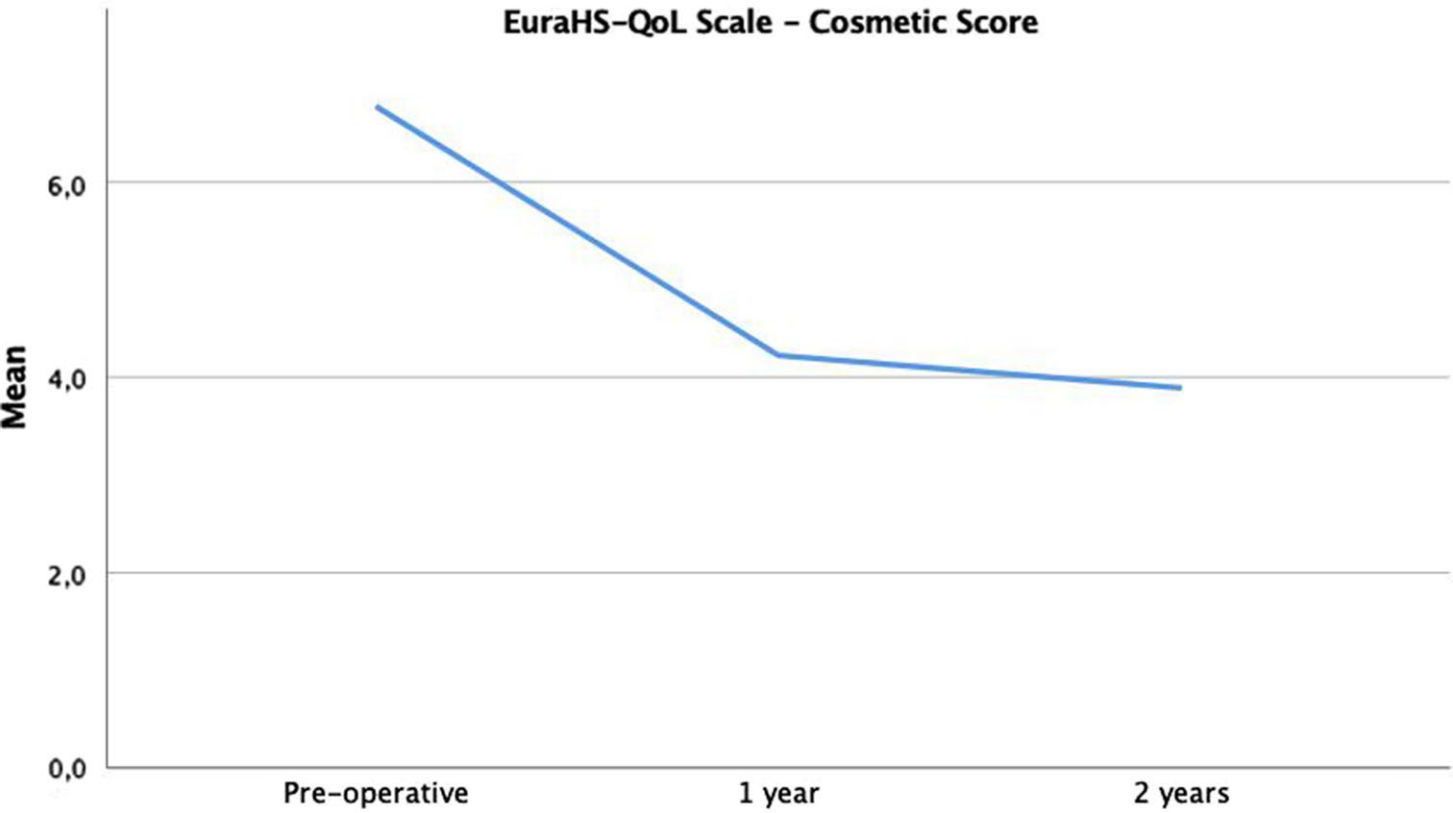
The evolution over time of EuraHS-QoL of cosmetic domain of is shown

EuraHS-QoL scores over time are shown (Figs. 2, 3, 4). The differences were statistically significant between preoperative and 1-year scores for pain (*p* = 0.05; moderate correlation) and restriction (*p* = 0.01; very strong correlation) domains. The difference was also was statistically significant in all three domains between 1 and 2 postoperative years (pain, *p* = 0.01; restriction, *p* = 0.04; cosmetic, *p* = 0.01; very strong correlation).

## Discussion

This study described the association of ACS and PCS techniques for AWR, in exceptional surgical circumstances. The impossibility of closing posterior or anterior layers were the two main reasons for adding the detachment of the external oblique muscle to the TAR (Table 6). In our experience, after the lateral incision on the posterior rectus sheath, we perform an extended dissection in the retromuscular preperitoneal plane from the central tendon of the diaphragm to the Cooper’s ligaments, and from the psoas–quadratum lumborum of one side, to the contralateral homonymous muscles. However, despite this extensive dissection and patient optimization with botulinum injections and pneumoperitoneum, the impossibility of closing the posterior layer, composed of the remaining peritoneal sac and posterior rectus sheath, may occur. This situation may result from a lack or obliteration of these layers because of previous surgeries, scarred tissue or previous mesh infections. In these circumstances, the use of omentum, or absorbable meshes have been described [16, 29, 30]. When the viscera cannot be returned to the abdomen, another surgical possibility is to reduce intra-abdominal volume by resection, in the following order, omentum, retroperitoneal lipomatosis or right hemicolectomy [31]. However, this last resection may lead to a postoperative anastomotic dehiscence, in a potentially increased abdominal pressure environment that could be disastrous. To prevent this risk, adding an external oblique muscle release to the TAR could be a possibility in some extreme cases, as observed in one of our patients. In this case, the return of abdominal content before completely closing posterior layer resulted in an increased in abdominal pressure and respiratory parameters. The addition of an external oblique release provided enough volume, improving the respiratory parameters.

**Table 6:** Possible surgical solutions when midline cannot be completely closed after a TAR

	Possible surgical solutions
Reasons for not closing posterior layer^a^	
Lack or obliteration of layer, mesh removals	Omentum interposition [29] Bridge with absorbable mesh [30]
Impossibility of viscera to return to the abdominal cavity Intra-abdominal hypertension Non tolerated increase of respiratory pressures	Visceral resection [31] External oblique release (present series)
Reasons for not closing anterior layer^b^	
Lack or obliteration of layer, mesh removals Scarred tissues, fibrosis, retracted muscles with very wide gap Intra-abdominal hypertension Non tolerated increase of respiratory pressures	Visceral resection [31] Myofascial flaps [32] Abdominal wall transplant [33] Abdominal wall expanding system [35] Bridged repair [21] External oblique release (present series) Staged procedure [36]

^a^Posterior layer is considered the rest of posterior rectus sheath and peritoneum on both sides of the abdomen

^b^Anterior layer is the anterior rectus sheath that insert on linea alba on both sides of the abdomen

The other surgical possibility after a TAR in challenging situations, is the impossibility of closing the anterior layer (linea alba). This layer usually consists of the rest of a retracted anterior aponeurosis of the rectus sheath that cannot be approximated. There are several reasons why a surgeon cannot perform closure of an anterior layer, despite a proper TAR (Table 6). Previous scars, infected meshes and the massive size of the defect could make it mechanically impossible to close. Also, too much tension on the abdomen may cause intra-abdominal hypertension or respiratory insufficiency.

Surgical solutions have been described to deal with these conditions: myofascial flaps, abdominal wall transplants, abdominal wall expanding systems, bridged repairs, staged repairs and in this study, adding an ACS to a PCS. Myofascial flaps have been used for AWR to cover significant defects in the abdomen and skin [32]. However, we agree that pedical or free flaps only provide soft tissue coverage of the intra-abdominal content [21]. Abdominal wall transplantation may also offer viscera protection, however, these procedures are complicated and involve immunosuppression therapy that impairs wound healing, and may facilitate infection and other long-term side effects [33]. The use of an abdominal wall expanding system has also been proposed in reducing, intraoperatively, the distance of the fascial defect, [34, 35]. There is very limited experience in  this method. Another possibility is to perform a temporary abdominal wall closure, using a mesh that is progressively excised to allow definite closure of the abdomen with a component separation [36]. In this initial report of staged repair, an average of six interval operations were necessary before the final operation. We consider that this staged surgery could be an option when increases in intra-abdominal pressure or respiratory insufficiency are primary concerns.

The other possibility is the use of a bridged repair after the TAR, as previously described [16, 20, 21, 37]. When a TAR is performed, and the anterior layer is not entirely closed, synthetic mesh is secured at the edges of the anterior layer. In a recent study of 77 patients with bridged repairs, patient-reported outcomes were favorable [21]. However, these authors also described a very high recurrence rate of nearly 50%. High incidences of recurrences have also been reported when bridging with biological meshes [38, 39]. Therefore, we advocate full reconstruction of the midline as a relevant component in complex AWR. This poses the question; what approach is ideal in these challenging cases? A TAR with a bridged repair or a TAR adding an ACS? The answer will depend on the size and location of the surface to be bridged.

Combining external oblique muscles and TAR is considered dangerous, because of the potential instability to the abdominal wall, as the only lateral muscle that remains anatomically attached to its anatomical insertions is the internal oblique muscle. However, some anatomical concepts require thoughtful review. Despite the TAR, the TA muscle still maintains its attachment to the internal oblique muscle all along its surface and, inferiorly, it also contributes to the anterior rectus sheath below the linea arcuata. In the TAR, we only release the transversus abdominis from its anatomical insertion in the posterior rectus sheath. After the TAR, we do not observe the retraction of the TA muscle that is developed when the external oblique is released, due to the strong interconnections between the internal oblique and transversus where the neurovascular bundles from the intercostal nerves are coming. Also, the size of the synthetic mesh we currently use in the TAR, overlaps the release of the external oblique muscle and this way, we cover the possibility of a subsequent defect or bulging in that area (Fig. 1). Furthermore, it is possible to reinsert the TA muscle into the synthetic mesh, to avoid the partial disconnection of the TA [17]. It must be remembered that positive results were published for TAR after recurrences of ACS techniques [13]. In a recent report, 29 patients were treated with a combination of ACS and PCS by the release of external oblique muscle through a debatable posterior access between neurovascular bundles [40].

**Fig. 5:**
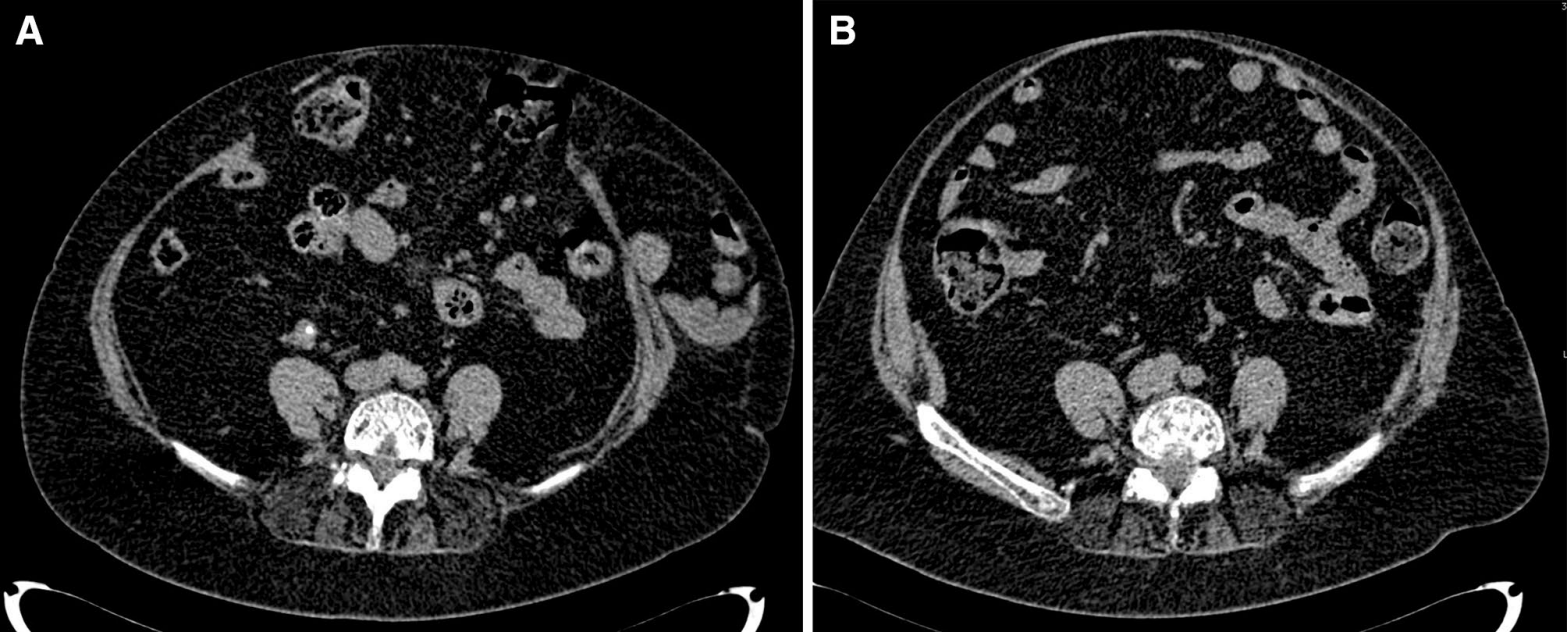
**a** Preoperative CT scan of an old lady with a midline defect 30 cm long and maximum 20 cm width. **b** Control CT scan in Valsalva at 18 postoperative months of the patient **a** after AWR with the combination of TAR and external oblique release. A weak but continent abdominal wall is observed

**Fig. 6:**
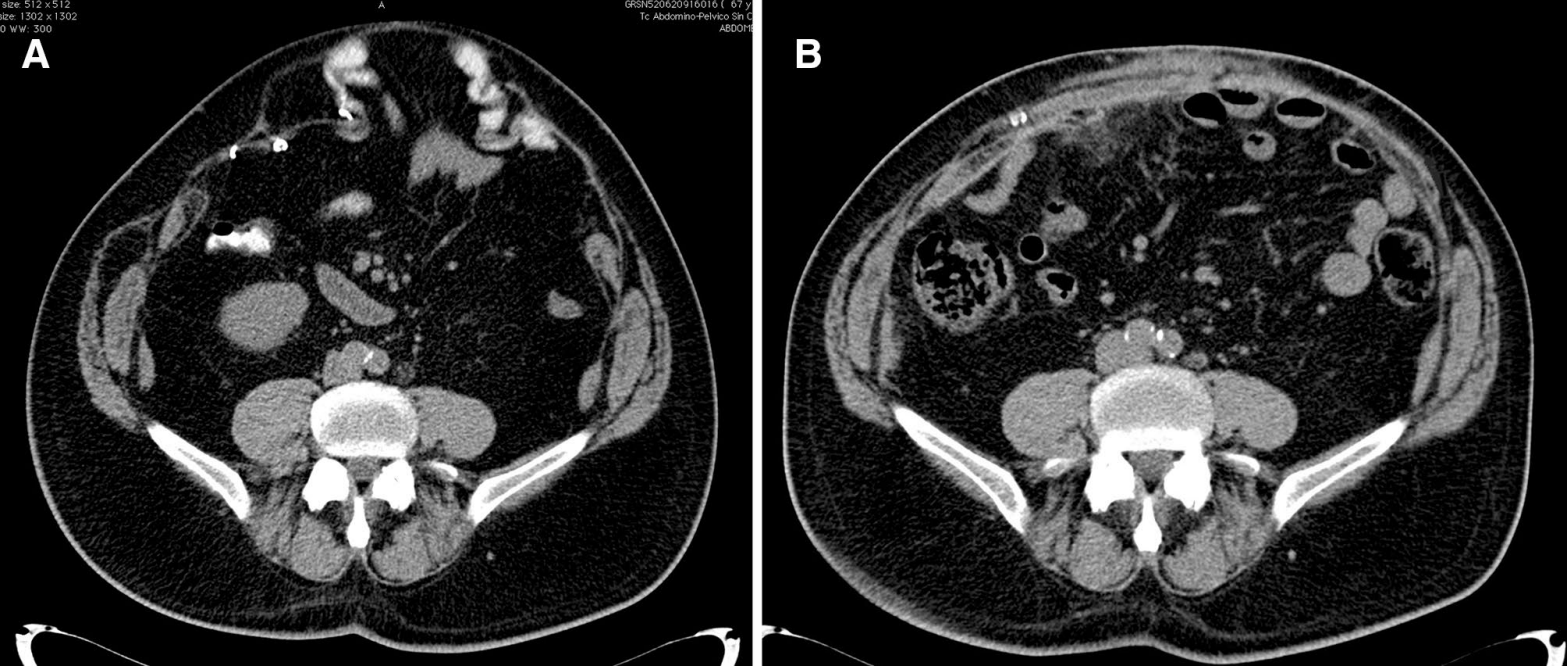
**a** Preoperative CT scan of a 58 years old man with a midline defect 28 cm long and maximum 22 cm width. **b** Control CT scan without Valsalva at 4 postoperative months of the patient **a** after AWR with the combination of TAR and external oblique release

We do not want to encourage surgeons to perform ACS and PCS combinations, but surgical possibilities under challenging settings must be debated. There is no need to remember the exceptional circumstances of this cohort of patients that associate severe comorbidities, multiple recurrences, big size of defects, and loss of domain (Figs. 5, 6). When dealing with these situations, the main objective of AWR is to provide abdominal wall continence. Combining ACS and TAR may provide closure of the abdomen and restore the connection between both sides of the abdominal wall. This continence was reflected by satisfactory patient-reported outcomes in our study. This objective was particularly achieved for the youngest patient in our cohort, as he is now playing regular sports, after his AWR. In our opinion, these difficult cases should be treated in dedicated centers of AWR with enough level of expertise.

In our cohort, associated wound morbidity was high. It is superior to our reported outcomes of AWR [16], and those from the largest series of TAR [14]. We do not get lower SSO and SSOPI published in similar larger series [21], despite adequate patient optimization. The only difference in our cohort was that we had significantly more patients with panniculectomy, which could partially explain our higher morbidity. Although it is a short cohort of patients, the long-term results obtained and the positive patient-reported outcomes are encouraging.

## Study limitations

Our study had limitations. With our small number of cases, it was difficult to draw definitive conclusions on the management of these highly complex repairs. We have not made comparisons with other surgical options, or other conservative non-operating approaches. However, a prospective group for comparison will be difficult, as the circumstances and etiopathogenesis that generate these complicated abdominal wall defects are infrequent and variable. Although all patients were followed-up clinically, a control CT scan was made available in only 66% of patients; therefore, a radiological recurrence could have been clinically missed. Although we have a mean follow-up of more than 2 years, a longer follow-up would be ideal in confirming surgical durability and stability.

In conclusion, combining external oblique release with TAR may be considered a surgical alternative in addressing demanding surgical situations, where defect complexity and patient characteristics do not allow complete midline reconstruction. While the approach is associated with high morbidity and a long hospital recovery, long-term results and patient-reported outcomes are reassuring.
