# Correction to: Combining anterior and posterior component separation for extreme cases of abdominal wall reconstruction

**DOI:** 10.1007/s10029-020-02343-y

**Published:** 2020-11-23

**Authors:** J. Lopez-Monclus, J. Muñoz-Rodríguez, C. San Miguel, A. Robin, L. A. Blazquez, M. Pérez-Flecha, N. Rupealta, M. A. Garcia-Urena

**Affiliations:** 1 Henares University Hospital (Coslada, Madrid), Faculty of Health Sciences, Francisco de Vitoria University, Carretera Pozuelo-Majadahonda km. 1,800, 28223 Pozuelo de Alarcón, Spain; 2 Puerta de Hierro University Hospital, Majadahonda, Madrid, Spain; 3 Ramón y Cajal University Hospital, Madrid, Spain

## Correction to: Hernia 10.1007/s10029-020-02152-3

The article Combining anterior and posterior component separation for extreme cases of abdominal wall reconstruction, written by J. Lopez-Monclus, J. Muñoz-Rodríguez, C. San Miguel, A. Robin, L. A. Blazquez, M. Pérez-Flecha, N. Rupealta and M. A. Garcia-Urena, was originally published Online First without Open Access. After publication in volume 24, issue 2, pages 369–379 the author decided to opt for Open Choice and to make the article an Open Access publication. Therefore, the copyright of the article has been changed to © The Author(s) 2020 and the article is licensed under a Creative Commons Attribution 4.0 International License, which permits use, sharing, adaptation, distribution and reproduction in any medium or format, as long as you give appropriate credit to the original author(s) and the source, provide a link to the Creative Commons licence, and indicate if changes were made. The images or other third party material in this article are included in the article’s Creative Commons licence, unless indicated otherwise in a credit line to the material. If material is not included in the article’s Creative Commons licence and your intended use is not permitted by statutory regulation or exceeds the permitted use, you will need to obtain permission directly from the copyright holder. To view a copy of this licence, visit http://creativecommons.org/licenses/by/4.0/.

The original article has been corrected.

